# Management of Lambda-Cyhalothrin Poisoning in a North Indian Healthcare Setup: A Rare Case

**DOI:** 10.7759/cureus.32746

**Published:** 2022-12-20

**Authors:** Sourav Sudan, Navjot Kaur, Rishabh Taneja, Navjot Kaur, Prachi Mehmi

**Affiliations:** 1 Internal Medicine, Government Medical College, Rajouri, Rajouri, IND; 2 Emergency Department, Sadbhavna Medical and Heart Institute, Patiala, IND; 3 Medical School, Government Multi-Specialty Hospital, Chandigarh, IND; 4 Graduate Medical Education, Adesh Institute of Medical Sciences and Research, Bathinda, IND; 5 Internal Medicine, Adesh Institute of Medical Sciences and Research, Bathinda, IND

**Keywords:** lambda-cyhalothrin, agricultural insecticide, organophosphates, pyrethroids, poisoning

## Abstract

Agricultural product (insecticides and pesticides) poisoning is common in the rural Indian setup, and in most cases, it occurs due to suicidal attempts rather than accidental intake. Furthermore, most cases of agrochemical poisoning are organophosphate poisoning rather than other less commonly used pesticides. Lambda-cyhalothrin is a less commonly used insecticide in India, and there have been a few instances in the literature where lambda-cyhalothrin poisoning and its subsequent management have been described. In this case report, we describe accidental lambda-cyhalothrin poisoning in a 26-year-old female and its management at our center.

## Introduction

With the increasing availability of insecticides/pesticides in rural India, cases of self-poisoning have risen steeply [1]. Similarly, on a global scale, the problem has worsened with approximately 300,000 annual deaths due to poisoning, the majority of which occur due to insecticide and pesticide intake [2].

The majority of cases of insecticide/pesticide poisoning in central and south India are due to organophosphates, while those in the northern parts of India are due to aluminum phosphide poisoning [3,4]. Cases of pyrethroid, carbamate, and organochlorine poisoning are less common [1].

Lambda-cyhalothrin is a synthetic pyrethroid, and its use in agriculture for insect and pest control has recently increased, linking it to various adverse effects [5]. Studies have linked exposure to lambda-cyhalothrin to endocrinological disorders, disruptions in the reproductive system, and even cancers [6]. Here, we report a case of accidental ingestion of lambda-cyhalothrin in a 26-year-old female who presented at our center with hemodynamic instability and respiratory distress. The patient was mechanically ventilated and given supportive treatment before her discharge, three days later.

## Case presentation

A 26-year-old female presented at our center eight hours after ingesting an unknown insecticide. Eight hours prior to the presentation, she accidentally ingested the insecticide while manipulating a spraying device and developed intractable vomiting 20 minutes later. After informing her family, she was rushed to a local healthcare center where the staff treated her for organophosphate poisoning as the family did not specify the insecticide ingested.

Her vitals upon admission at that center were as follows: blood pressure (BP) of 90/60 mmHg, pulse rate (PR) of 62 beats per minute (bpm), and SaO_2_ of 96%. Gastric lavage was performed, and she was administered the standard treatment for organophosphate poisoning, i.e., intravenous (IV) fluids, injection pralidoxime (PAM) 1 g IV eight hourly, and injection atropine 20 mg IV stat, followed by 10 mg in 100 mL normal saline (NS) over the next two hours.

The patient’s condition continued to deteriorate despite the treatment, and her vitals in the next two hours were as follows: BP of 100/60 mmHg, PR of 110 bpm, and SaO_2_ of 88% (on room air). Furthermore, the patient became irritable and had an altered mental sensorium. She was subsequently referred to our center.

Upon arrival at our center, the patient was unconscious with a Glasgow Coma Scale (GCS) score of 4/15, BP of 78/42 mmHg, PR of 85 bpm, and SaO_2_ of 92% on high-flow oxygen. Considering her low GCS score, endotracheal intubation was performed, and mechanical ventilation was commenced.

After much probing, the family brought the bottle of the insecticide, which revealed that the compound ingested was lambda-cyhalothrin, an uncommon insecticide. The patient was then commenced on noradrenaline 2 mg in 500 mL NS at 30 mL/hour. Injection hydrocortisone 100 mg BD and injection pheniramine 2 mL IV stat were also administered.

All baseline investigations were ordered, and she was kept sedated and on mechanical ventilation for the next two days with continuous monitoring. Noradrenaline was infused for hemodynamic support, and her vitals were monitored regularly (Tables 1, 2).

**Table 1:** Basic blood workup during the three days of inpatient admission. SGOT = serum glutamic-oxaloacetic transaminase; SGPT = serum glutamic pyruvic transaminase

Parameters (normal)	Day one	Day two	Day three
Serum urea (10–50 mg/dL)	46.0 mg/dL	42.4 mg/dL	38.0 mg/dL
Serum creatinine (0.8–1.5 mg/dL)	1.3 mg/dL	1.2 mg/dL	1.4 mg/dL
SGOT (4–40 IU/L)	30.4 IU/L	38.0 IU/L	36.6 IU/L
SGPT (3–35 IU/L)	35.7 IU/L	38.6 IU/L	40.0 IU/L
Blood sugar random (up to 150 mg/dL)	116.0 mg/dL	124 mg/dL	118 mg/dL
Serum bilirubin (0.2–1.2 mg/dL)	0.95 mg/dL	0.9 mg/dL	1.1 mg/dL

**Table 2:** Arterial blood gas values over three days of inpatient admission. ABG = arterial blood gas; pCO_2_ = partial pressure of carbon dioxide; pO_2_ = partial pressure of oxygen; SaO_2_ = arterial oxygen saturation; sNa^+^ = serum sodium; sK^+^ = serum potassium, sCa^+^ = serum calcium; sCl^-^: serum chloride; sHCO_3_^-^ = serum bicarbonate

ABG parameters (normal values)	Day one	Day two	Day three
pH (7.350–7.450)	7.315	7.281	7.404
pCO_2_ (35.0–45.0 mmHg)	37.9 mmHg	45.4 mmHg	31.5 mmHg
pO_2_ (83.0–108 mmHg)	95.2 mmHg	100 mmHg	98 mmHg
SaO_2_ (95.0%–99.0%)	99.9%	97.8%	99%
sNa^+^ (135–145 mmol/L)	146 mmol/L	154 mmol/L	144 mmol/L
sK^+^ (3.5–5.5 mmol/L)	3.7 mmol/L	3.3 mmol/L	3.0 mmol/L
sCa^+^ (1.15–1.29 mmol/L)	0.84 mmol/L	0.92 mmol/L	1.00 mmol/L
sCl^-^ (96–106 mmol/L)	109 mmol/L	112 mmol/L	114 mmol/L
sHCO_3_^-^ (18–24 mmol/L)	19.9 mmol/L	26.2 mmol/L	19.3 mmol/L

The patient was successfully extubated on day three and placed on a high-flow mask, which was subsequently removed after six hours. Her vitals were stable, and after a few hours of monitoring, she was moved to the medical ward. She was monitored for two more days and discharged on oral proton pump inhibitors. Follow-up has been uneventful.

## Discussion

Lambda-cyhalothrin belongs to a class of chemical compounds known as pyrethroids, which are of two types, namely, type 1 (which includes compounds such as allethrin and permethrin) and type 2 (which includes lambda-cyhalothrin and related compounds) pyrethroids. The symptoms of toxicity of the two types differ, with type 1 pyrethroid toxicity inducing mild symptoms such as fine tremors and hyperexcitability (type 1 syndrome or T syndrome) [7] and type 2 pyrethroid toxicity inducing moderate-to-severe symptoms such as incoordination, choreoathetosis, seizures, hypersalivation, and direct damage to the skeletal and cardiac musculature (type 2 syndrome) [7].

The major life-threatening manifestations of pyrethroid poisoning include seizures, pulmonary edema, and hemorrhage [8]. The mechanism of toxicity is attributed to changes in ionic conduction across the various sodium, calcium, and chloride channels and to direct oxidative damage to tissues [9,10].

Because there is no known antidote for lambda-cyhalothrin poisoning, management is mainly supportive. Immediate steps are similar to those of other cases of poisoning and include removal from the environment of exposure and decontamination.

At the medical center, the airway, breathing, and circulation should be secured first. If there are associated seizures, as seen in type 2 syndrome, they should be managed with proper antiepileptic therapy. There are reports showing that benzodiazepines are more effective than barbiturates in controlling seizures caused by pyrethroid toxicity [11]. Furthermore, hemodynamic instability, if present, should be managed with vasopressors, as we did for this patient.

Research regarding the antidote for pyrethroid toxicity is ongoing, and animal studies have shown a possible therapeutic role of methocarbamol; however, this has not been studied in human subjects [12].

## Conclusions

This case report describes acute lambda-cyhalothrin toxicity and how it can present with hemodynamic instability, respiratory distress, and neurological manifestations. As seen in this case, treatment is primarily supportive and symptomatic. Mechanical ventilation may be required, and a low threshold should be kept for invasive mechanical ventilation. The outcome was favorable in our patient, and the same can be expected in young populations.
